# Reduced Volume and Faster Infusion Rate of Activated Prothrombin Complex Concentrate: A Phase 3b/4 Trial in Adults with Hemophilia A with Inhibitors

**DOI:** 10.1055/s-0044-1787781

**Published:** 2024-07-08

**Authors:** Bülent Zülfikar, Johnny Mahlangu, Salim Mohamed Nekkal, Cecil Ross, Noppacharn Uaprasert, Jerzy Windyga, Carmen Escuriola Ettingshausen, Bettina Ploder, Aurelia Lelli, Hanna T. Gazda

**Affiliations:** 1 Hereditary Bleeding Disorders Unit in Oncology Institute, Istanbul University, Istanbul, Turkey; 2 Department of Molecular Medicine and Haematology, Faculty of Health Sciences, University of the Witwatersrand and NHLS, Johannesburg, South Africa; 3 CHU Isaad Hassani, Beni Messous, Algiers, Algeria; 4 St John's Medical College Hospital, Bangalore, India; 5 Department of Medicine, Faculty of Medicine, Chulalongkorn University and King Chulalongkorn Memorial Hospital, Thai Red Cross Society, Bangkok, Thailand; 6 Center of Excellence in Translational Hematology, Faculty of Medicine, Chulalongkorn University and King Chulalongkorn Memorial Hospital, Thai Red Cross Society, Bangkok, Thailand; 7 Department of Hemostasis Disorders and Internal Medicine, Laboratory of Hemostasis and Metabolic Diseases, Institute of Hematology and Transfusion Medicine, Warsaw, Poland; 8 Haemophilia Centre Rhein Main, Frankfurt-Mörfelden, Germany; 9 Baxalta Innovations GmbH, a Takeda company, Vienna, Austria; 10 PDT R&D Global Medical Affairs, Takeda Pharmaceuticals International AG, Zurich, Switzerland; 11 Takeda Development Center Americas, Inc., Cambridge, Massachusetts, United States

**Keywords:** aPCC, safety, treatment burden, hemophilia with inhibitors

## Abstract

**Background** Activated prothrombin complex concentrate (aPCC) is indicated for bleed treatment and prevention in patients with hemophilia with inhibitors. The safety and tolerability of intravenous aPCC at a reduced volume and faster infusion rates were evaluated.

**Methods** This multicenter, open-label trial (NCT02764489) enrolled adults with hemophilia A with inhibitors. In part 1, patients were randomized to receive three infusions of aPCC (85 ± 15 U/kg) at 2 U/kg/min (the approved standard rate at the time of the study), in a regular or 50% reduced volume, and were then crossed over to receive three infusions in the alternative volume. In part 2, patients received three sequential infusions of aPCC in a 50% reduced volume at 4 U/kg/min and then at 10 U/kg/min. Primary outcome measures included the incidence of adverse events (AEs), allergic-type hypersensitivity reactions (AHRs), infusion-site reactions (ISRs), and thromboembolic events.

**Results** Of the 45 patients enrolled, 33 received aPCC in part 1 and 30 in part 2. In part 1, 24.2 and 23.3% of patients with regular and reduced volumes experienced AEs, respectively; 11 AEs in eight patients were treatment related. AHRs and ISRs occurred in four (12.1%) and two (6.1%) patients, respectively. In part 2, 3.3 and 14.3% of patients with infusion rates of 4 and 10 U/kg/min experienced AEs, respectively; only one AE in one patient was treatment related; no AHRs or ISRs were reported. Most AEs were mild/moderate in severity. Overall, no thromboembolic events were reported.

**Conclusions** aPCC was well tolerated at a reduced volume and faster infusion rates, with safety profiles comparable to the approved regimen.

## Introduction

Congenital hemophilia is a rare, X-linked bleeding disorder characterized by a deficiency in coagulation factor VIII (FVIII; hemophilia A) or factor IX (hemophilia B). 1 The partial or total absence of these factors results in spontaneous bleeding episodes, which occur primarily in joints, muscles, and, less commonly, soft tissues, as well as excessive bleeding following trauma or surgery. 1

Patients with hemophilia A can be treated with clotting factor concentrates or nonfactor therapies. 1 Approximately 30% of patients with severe hemophilia A treated with clotting factor concentrates develop antibodies, also referred to as inhibitors, against these products, which results in their neutralization and loss of function. 1 2 The development of inhibitors remains the most serious complication associated with hemophilia treatment. 3 4 Resistance to factor replacement therapy predisposes patients to increased morbidity and mortality, including an increased need for orthopedic surgery and a reduced health-related quality of life, compared with patients without inhibitors. 1 5 6

Bypassing agents (BPAs), such as activated prothrombin complex concentrate (aPCC) and recombinant activated factor VII (rFVIIa), can be used for the management of acute bleeds or surgeries in patients with hemophilia with inhibitors. 1 7 Both aPCC and rFVIIa can also be used for prophylaxis to prevent bleeding complications in these patients, although at the time of writing, only aPCC is U.S. Food and Drug Administration-approved for this indication. 8 9 10 11 12 The nonfactor therapy emicizumab is also approved for bleed prevention in patients with hemophilia A with or without inhibitors, but not for the treatment of acute bleeds. 1

aPCC is indicated for controlling and preventing bleeding episodes, perioperative management, and routine prophylaxis to prevent or reduce the frequency of bleeding episodes in patients with congenital hemophilia with inhibitors. 12 The approved dosage for routine prophylaxis is 85 U/kg every other day (dose strength [regular volume of sterile water]: 500 U [10 mL], 1,000 U [20 mL], or 2,500 U [50 mL] = 50 U/mL) at the standard infusion rate, which was 2 U/kg/min at the time of this study. 12 13 Despite this, a study of real-world aPCC use in patients with congenital hemophilia with inhibitors found that, in routine clinical practice, patients received aPCC at a faster mean infusion rate of 3.8 U/kg/min. 13 When considered together with the time-consuming nature of frequent intravenous infusions, 1 these real-world findings highlight the need for an optimized approach to administering aPCC in patients with hemophilia with inhibitors.

In this trial, we evaluated the tolerability and safety of intravenously administered aPCC in a 50% reduced volume at an infusion rate of 2 U/kg/min and at increased infusion rates of 4 and 10 U/kg/min in patients with hemophilia with inhibitors.

## Methods

### Study Design and Procedures

This was a two-part, phase 3b/4, prospective, multicenter, open-label, randomized, crossover trial in adults with hemophilia with inhibitors (ClinicalTrials.gov identifier: NCT02764489; EudraCT identifier: 2015-005781-39). The study was conducted at 18 study centers in eight countries in Africa, Asia, and Europe, and patients were enrolled for up to 11 weeks. An independent ethics committee reviewed and approved the final protocol and four amendments. All patients provided written informed consent to participate in the study, at which point they were considered enrolled. The study was conducted in accordance with the Declaration of Helsinki.

**Fig. 1:**
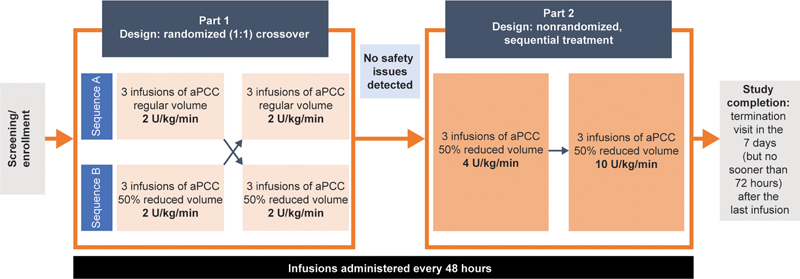
Study design. Patients were required to have received at least two of the three infusions in each sequence in part 1 to be eligible for part 2. aPCC was supplied at 85 ± 15 U/kg and reconstituted in sterile water for infusion. aPCC, activated prothrombin complex concentrate.

In part 1, patients were centrally randomized (1:1) to receive either three infusions of aPCC (85 ± 15 U/kg) reconstituted in a regular volume of sterile water followed by three infusions of aPCC in a 50% reduced volume (sequence A), or three infusions of aPCC (85 ± 15 U/kg) reconstituted in a 50% reduced volume followed by three infusions of aPCC in a regular volume (sequence B; Fig. 1 ). All infusions in part 1 were given at a rate of 2 U/kg/min. In part 2, patients who received at least two of the three infusions in each sequence in part 1 and who reported no safety issues received three sequential (nonrandomized) infusions of aPCC (85 ± 15 U/kg) in a 50% reduced volume infused at 4 U/kg/min and then subsequently at 10 U/kg/min if no safety issues were detected. Infusions were administered every 48 hours during both parts of the study using intravenous infusion pumps to standardize the administration of aPCC. After each infusion, patients were observed for at least 30 minutes at the study site. There was no washout period before the study started, between parts 1 and 2, or between any of the infusions. Given that the study included changes to the infusion volume and rate, blinding was not feasible.

Patients were considered to have completed the study when they had completed all procedures according to the protocol. Study completion or termination visits were performed in the 7 days (but no sooner than 72 hours) after the final protocol-defined infusion for patients who completed the study or after the last infusion for those who discontinued treatment, respectively.

### Patients

Patients were eligible for this study if they were aged 18 to 65 years and had hemophilia A or B of any severity, with a documented history (≥3 months) of inhibitors (≥0.6 Bethesda units) requiring the use of BPAs (aPCC or rFVIIa) before screening (inhibitor levels were tested at screening if no documented history was available). In addition, patients were required to have a negative hepatitis C virus result or a positive hepatitis C result with stable liver disease; and a negative human immunodeficiency virus (HIV) result or a positive HIV result with stable disease and a cluster of differentiation 4 count of at least 200 cells/mm ^3^ at screening. Critical exclusion criteria were the following: known hypersensitivity to aPCC or any of its components; advanced liver disease; planned elective surgery during participation in this study (excluding minor procedures that would not need preventive bleeding treatments, such as exchanges of peripherally inserted central catheters); platelet count of less than 100,000/µL; receiving emicizumab for bleed prevention; clinical or laboratory evidence of disseminated intravascular coagulation; and history or current evidence of thromboembolic events.

Concomitant medications permitted during the study included any medication deemed necessary by the patient's physician to treat or prevent a medical condition. Antifibrinolytics were prohibited within approximately 6 to 12 hours of administration of aPCC. Concomitant use of rFVIIa was avoided, except when used to treat breakthrough bleeding that did not respond to aPCC. Use of emicizumab, any immunomodulatory agent, or any investigational drug or device was not permitted. Additional inclusion and exclusion criteria are provided in Supplementary Material S1 .

### Outcome Measures

The primary outcome measures were the occurrence of any adverse events (AEs), allergic-type hypersensitivity reactions (AHRs), infusion-site reactions (ISRs), thromboembolic events, AEs leading to study discontinuation, and changes in vital signs and laboratory parameters that were considered AEs. Each AE was categorized according to treatment (infusion volume and rate) and evaluated by the investigator for its seriousness, severity, and causal relationship to aPCC or study procedures. AEs could be considered related to both aPCC administration and study procedures. AEs were coded according to the Medical Dictionary for Regulatory Activities version 24.1.

Exploratory outcome measures included monitoring of pre- (≤60 minutes before) and postinfusion (0.5, 1, 2, 6, 8, and 12 hours after) activity of coagulation factor II (FII). Patient treatment preferences were evaluated after infusions 3, 6, 9, and 12/up to study completion or the study termination visit, using the 9-item Treatment Satisfaction Questionnaire for Medication (TSQM-9) 14 and a patient preference questionnaire. The TSQM-9 is a validated instrument developed to assess patient satisfaction with medication in three domains: effectiveness, convenience, and global satisfaction. TSQM-9 scores for each of the domains were graded on a scale from 1 to 100, with higher scores indicating greater satisfaction. 14 The patient preference questionnaire consisted of two questions with a 7-point Likert satisfaction scale (from “extremely satisfied” to “extremely dissatisfied”), which assessed patient satisfaction with infusion times and ease of fitting the treatment into their schedules. All responses were recorded in case report forms and presented in summary figures.

### Statistical Analyses

A planned sample size of at least 24 evaluable patients was determined by considering available patients and was not based on statistical power calculations. Evaluable patients were all patients who received at least two infusions per sequence in part 1 and at least two infusions per infusion rate in part 2. To allow for a nonevaluable rate of 25%, we aimed to enroll at least 32 patients.

All statistical analyses for this study were descriptive. Patients were evaluated according to the treatment (infusion volume and rate) received. The safety analysis set included all patients who received at least one dose of aPCC. All safety analyses, as well as the analyses based on the TSQM-9 and patient preference questionnaire were performed on the safety analysis set. The full analysis set comprised all patients who were randomized to study treatment in part 1, received at least one dose of aPCC, had a pre-infusion FII level measurement, and had at least two postinfusion results for the same administration of infusion 1, 3, 4, 6, 9, or 12. The analyses based on FII activity levels were performed on the full analysis set.

Primary outcome measures were presented in summary tables. Responses to global satisfaction and convenience scores based on the TSQM-9 were presented in summary figures. Owing to uncertainty around prior dosing or steady state and the unknown clinical relevance of FII as a marker for aPCC, FII activity levels were analyzed as a standardized increase from the corresponding predose levels. The standardized increase was calculated using the following formula:



where *C _t_* = the FII level at time point *t* , *C*_pre_ = the corresponding predose level, and dose = the weight-adjusted dose.

The time-averaged standardized increase (TASI) in FII activity levels up to 48 hours after infusion was calculated based on the area under the curve (AUC) of the standardized increase up to 48 hours as:



For calculation of AUC _0–48h_ , the FII activity level at exactly 48 hours was log-linearly interpolated from the two nearest sampling points from the same FII activity level profile for calculating the standardized increase at exactly 48 hours.

In part 1 of the study, a linear mixed-effects model was used, modeling the sequence, infusion number, and volume as fixed effects, and patient nested within the sequence as a random effect, to estimate a potential impact of infusion volume (regular and reduced) at an infusion rate of 2 U/kg/min on the TASI in FII activity levels up to 48 hours after infusion. For part 2 of the study, a linear mixed-effects model was used, modeling the infusion rate as a fixed effect and the patient as a random effect, to estimate the potential impact of infusion rate (4 and 10 U/kg/min) at the 50% reduced volume on the TASI in FII activity levels up to 48 hours after infusion.

Treatment adherence regarding the infusion volume was calculated as the number of infusions within 10% of the required infusion volume divided by the total number of administered infusions. Treatment compliance regarding the infusion rate was calculated as the number of infusions that were not interrupted and were within 10% of the planned infusion rate, divided by the total number of administered infusions that were not interrupted. Finally, treatment compliance regarding the infusion dose was calculated as the number of infusions within the recommended dose of 85 ± 15 U/kg, divided by the total number of administered infusions.

## Results

### Baseline Demographics and Clinical Characteristics

In total, 45 patients were screened and enrolled in the study between February 12, 2019, and December 27, 2021. Of these, 12 patients (26.7%) discontinued the study before receiving the first infusion of aPCC, mainly owing to screening failure ( Supplementary Fig. S1 ). Of the 33 patients who received at least one dose of aPCC, 30 (90.9%) completed part 1 of the study, of whom 28 (93.3%) completed part 2 of the study. Three patients discontinued the study during part 1 owing to either an AE ( *n* = 2; drug hypersensitivity and severe hypersensitivity) or patient withdrawal ( *n* = 1). Two patients prematurely discontinued the study in part 2, one owing to an AE of increased fibrin D-dimer and one based on the physician's decision ( Supplementary Fig. S1 ). Mean treatment compliance across both parts of the study regarding infusion volume, rate, and dose was generally over 90%, except for mean treatment compliance regarding the infusion rate of 2 U/kg/min at the regular volume (86.5%).

Baseline demographics and clinical characteristics are presented in Table 1 . The mean (standard deviation [SD]) age of the patients was 35.4 (11.9) years, and all the patients were male. The mean (SD) past annualized bleed rate was 8.5 (9.6). All the patients had congenital hemophilia A with inhibitors; in most cases, this was severe (87.9% [29/33]). Median FVIII inhibitor levels were 14.4 Bethesda units. The mean (SD) baseline FII activity level was 1.2 (0.2) IU/mL. A total of 17 patients (51.5%) had a negative result for hepatitis C and all the patients had a negative result for HIV. Medical history data were reported for 30 patients (90.9%). The most frequently reported conditions (in more than two patients) were hemophilic arthropathy ( *n* = 7, 21.2%), hemarthrosis ( *n* = 5, 15.2%), hypertension ( *n* = 5, 15.2%), and hepatitis C ( *n* = 4, 12.1%). Nine patients (27.3%) had received BPAs (aPCC or rFVIIa) in the 3 weeks before screening, and 23 patients (69.7%) had received BPAs in the 12 months before screening.

**Table 1:** Baseline demographics and clinical characteristics (safety analysis set)

Baseline demographic or characteristic	Total population ( *n* = 33)
Age, y, mean (SD)	35.4 (11.9)
Minimum, maximum	18, 60
Male sex, *n* (%)	33 (100)
Race, *n* (%)	
Asian	17 (51.5)
Indian	8 (47.1)
Other a	9 (52.9)
White	16 (48.5)
Ethnicity, *n* (%)	
Hispanic or Latino	2 (6.1)
Not Hispanic or Latino	31 (93.9)
Height, cm, mean (SD)	169.1 (7.7)
Weight, kg, mean (SD)	67.5 (12.9)
BMI, kg/m ^2^ , mean (SD)	23.6 (4.3)
Past annualized bleed rate, mean (SD)	8.5 (9.6)
Congenital hemophilia A, *n* (%)	33 (100)
Hemophilia type and severity, b *n* (%)	
Hemophilia A, moderate	4 (12.1)
Hemophilia A, severe	29 (87.9)
Karnofsky index score, %, mean (SD)	80.6 (9.0)
Factor II activity, IU/mL, mean (SD)	1.2 (0.2)
Hepatitis C, *n* (%)	
Positive	15 (45.5)
Negative	17 (51.5)
Indeterminate	1 (3.0)
Platelet count, 10 ^9^ /L, mean (SD)	261.5 (81.4)
HIV negative, *n* (%)	33 (100)

Abbreviations: BMI, body mass index; HIV, human immunodeficiency virus; *n* , number of patients; SD, standard deviation.

Note: Baseline is defined as the last nonmissing result before the first administration of activated prothrombin complex concentrate.

a Other patients were of Malaysian or Thai race.

b Moderate hemophilia A: factor VIII activity, 1 to 5%; severe hemophilia A: factor VIII activity, <1%. 1

### Primary Safety Outcomes

#### Summary of AEs

Overall, 33 AEs were reported in 15 patients (45.5%) during the study; of these, 12 AEs in nine patients (27.3%) were related to administration of aPCC. The maximum severity for all AEs was mild or moderate in most patients (80.0%; Table 2 ). A summary of AEs by system organ class is presented in Supplementary Table S1 . No thromboembolic events were reported in either part 1 or part 2 of the study.

**Table 2:** Summary of primary outcome measures (safety analysis set)

AE, *n* (%) [ *m* ]	Regular volume 2 U/kg/min ( *n* = 33)	50% reduced volume 2 U/kg/min ( *n* = 30)	50% reduced volume 4 U/kg/min ( *n* = 30)	50% reduced volume 10 U/kg/min ( *n* = 28)	Overall 2 U/kg/min (both volumes) ( *n* = 33)	Overall 50% reduced volume (all infusion rates) ( *n* = 30)	Overall ( *n* = 33)
Any a	8 (24.2) [14]	7 (23.3) [14]	1 (3.3) [1]	4 (14.3) [4]	13 (39.4) [28]	10 (33.3) [19]	15 (45.5) [33]
Maximum severity b							
Mild	3 (9.1)	3 (10.0)	1 (3.3)	2 (7.1)	4 (12.1)	5 (16.7)	6 (18.2)
Moderate	4 (12.1)	2 (6.7)	0 (0.0)	2 (7.1)	6 (18.2)	3 (10.0)	6 (18.2)
Severe	1 (3.0)	2 (6.7)	0 (0.0)	0 (0.0)	3 (9.1)	2 (6.7)	3 (9.1)
Leading to study discontinuation	2 (6.1) [2]	1 (3.3) [1]	0 (0.0) [0]	0 (0.0) [0]	3 (9.1) [3]	1 (3.3) [1]	3 (9.1) [3]
Considered an AHR	3 (9.1) [4]	1 (3.3) [1]	0 (0.0) [0]	0 (0.0) [0]	4 (12.1) [5]	1 (3.3) [1]	4 (12.1) [5]
Considered an ISR	2 (6.1) [2]	1 (3.3) [1]	0 (0.0) [0]	0 (0.0) [0]	2 (6.1) [3]	1 (3.3) [1]	2 (6.1) [3]
Considered a thromboembolic event	0 (0.0) [0]	0 (0.0) [0]	0 (0.0) [0]	0 (0.0) [0]	0 (0.0) [0]	0 (0.0) [0]	0 (0.0) [0]

Abbreviations: AE, adverse event; AHR, allergic-type hypersensitivity reaction; ISR, infusion-site reaction; *m* , number of events; *n* , number of patients.

a In the regular volume 2 U/kg/min group, no AE was reported in more than one patient. The most commonly reported AEs in the 50% reduced volume 2 U/kg/min group were arthralgia ( *n* = 3; *m* = 4) and headache ( *n* = 2; *m* = 2). In the 50% reduced volume 4 U/kg/min group, one patient had an AE of viral infection. In the 50% reduced volume 10 U/kg/min group, the most commonly reported AE was arthralgia ( *n* = 2; *m* = 2).

b Patients were counted once, considering the highest severity AE.

In part 1 of the study, AEs were reported in eight patients (24.2%) receiving the regular volume at 2 U/kg/min; none of these AEs was reported in more than one patient. A total of seven patients (23.3%) receiving the 50% reduced volume at 2 U/kg/min in part 1 experienced AEs; the most commonly reported AEs were arthralgia ( *n* = 3, 10.0%) and headache ( *n* = 2, 6.7%). In part 2 of the study (patients who had received at least two of the three infusions in each sequence in part 1 and who reported no safety issues), only one patient (3.3%) receiving the 50% reduced volume at 4 U/kg/min experienced an AE (viral infection [reported by the investigator as viral fever]; the patient received acetaminophen as treatment). Four patients (14.3%) receiving the 50% reduced volume at 10 U/kg/min in part 2 experienced AEs; the most commonly reported AE was arthralgia ( *n* = 2, 7.1%).

#### AEs Leading to Study Discontinuation

Three AEs leading to study discontinuation were reported in three patients (9.1%). These were all reported during part 1 of the study, in two patients (6.1%) receiving the regular volume at 2 U/kg/min (moderate drug hypersensitivity, considered possibly related to the study drug, and severe hypersensitivity, considered probably related to the study drug) and one patient (3.3%) receiving the 50% reduced volume at 2 U/kg/min (severe increased fibrin D-dimer, considered probably related to study drug).

#### AEs Considered as AHRs and ISRs

Five AEs considered to be AHRs were observed in four patients (12.1%) during the study. In part 1 of the study, three patients (9.1%) receiving the regular volume at 2 U/kg/min experienced four events (two mild, one moderate, and one severe), and one patient (3.3%) receiving the 50% reduced volume at 2 U/kg/min experienced one mild event. In part 2 of the study, no AEs considered to be AHRs were reported. A total of two patients (6.1%) in the study experienced three AEs considered to be ISRs. In part 1 of the study, two patients (6.1%) receiving the regular volume at 2 U/kg/min experienced two events (moderate drug hypersensitivity and mild injection-site swelling), and one patient (3.3%) receiving the 50% reduced volume at 2 U/kg/min experienced one event (moderate thrombophlebitis). No AEs considered to be ISRs were reported in part 2 of the study.

#### Changes in Laboratory Parameters and Vital Signs Considered as AEs

No AEs associated with clinical chemistry or hematology laboratory abnormalities were reported. Two patients experienced two AEs associated with changes in coagulation parameters (increased fibrin D-dimer, considered probably related to the study drug, and muscle hemorrhage, considered unlikely to be related to the study drug). Both AEs occurred when patients received the regular volume at 2 U/kg/min and the 50% reduced volume at 2 U/kg/min. The AE of increased fibrin D-dimer was observed when the patient was due to receive their fourth dose of aPCC (after receiving two doses and missing the third dose of aPCC in the regular volume at 2 U/kg/min, and before receiving their first dose of aPCC in the 50% reduced volume at 2 U/kg/min). The patient tested positive for severe acute respiratory syndrome coronavirus 2 (SARS-CoV-2) antibodies 11 days after the AE was reported; the patient discontinued the study 2 weeks after the positive test result. The outcome of the AE was unknown at the last follow-up. The AE of muscle hemorrhage was a psoas bleed that was observed 5 days after the patient received their first dose of aPCC (3 days after their last dose of aPCC); study treatment was interrupted and the patient recovered from the bleed. In addition, a vital sign abnormality of increased systolic pressure that was considered possibly related to treatment by the investigator was reported in one patient who received the 50% reduced volume at 10 U/kg/min.

### Additional Safety Outcomes

#### Serious AEs

Overall, no deaths were reported in either part 1 or part 2 of the study. Four serious AEs (SAEs) were reported in four patients (12.1%) during the study ( Table 3 ). In part 1 of the study, three patients (9.1%) receiving the regular volume at 2 U/kg/min experienced three events (epilepsy, drug hypersensitivity, and hypersensitivity), and one patient (3.3%) receiving the 50% reduced volume at 2 U/kg/min experienced one event (muscle hemorrhage). No SAEs were reported in part 2 of the study. Two patients who received the regular volume at 2 U/kg/min experienced two SAEs considered either possibly or probably related to aPCC administration (drug hypersensitivity and hypersensitivity, respectively).

**Table 3:** Summary of additional safety outcome measures (safety analysis set)

AE, *n* (%) [ *m* ]	Regular volume 2 U/kg/min ( *n* = 33)	50% reduced volume 2 U/kg/min ( *n* = 30)	50% reduced volume 4 U/kg/min ( *n* = 30)	50% reduced volume 10 U/kg/min ( *n* = 28)	Overall 2 U/kg/min (both volumes) ( *n* = 33)	Overall 50% reduced volume (all infusion rates) ( *n* = 30)	Overall ( *n* = 33)
Serious a	3 (9.1) [3]	1 (3.3) [1]	0 (0.0) [0]	0 (0.0) [0]	4 (12.1) [4]	1 (3.3) [1]	4 (12.1) [4]
Leading to death	0 (0.0) [0]	0 (0.0) [0]	0 (0.0) [0]	0 (0.0) [0]	0 (0.0) [0]	0 (0.0) [0]	0 (0.0) [0]
aPCC-related	4 (12.1) [7] b	4 (13.3) [4] c	0 (0.0) [0]	1 (3.6) [1] d	8 (24.2) [11]	5 (16.7) [5]	9 (27.3) [12]
Serious study drug-related	2 (6.1) [2]	0 (0.0) [0]	0 (0.0) [0]	0 (0.0) [0]	2 (6.1) [2]	0 (0.0) [0]	2 (6.1) [2]
Hypersensitivity	1 (3.0) [1]	0 (0.0) [0]	0 (0.0) [0]	0 (0.0) [0]	1 (3.0) [1]	0 (0.0) [0]	1 (3.0) [1]
Drug hypersensitivity	1 (3.0) [1]	0 (0.0) [0]	0 (0.0) [0]	0 (0.0) [0]	1 (3.0) [1]	0 (0.0) [0]	1 (3.0) [1]
Study procedure-related e	3 (9.1) [5]	4 (13.3) [4]	0 (0.0) [0]	0 (0.0) [0]	6 (18.2) [9]	4 (13.3) [4]	6 (18.2) [9]

Abbreviations: AE, adverse event; aPCC, activated prothrombin complex concentrate; *m* , number of events; *n* , number of patients.

a Serious AEs were drug hypersensitivity ( *n* = 1; *m* = 1), epilepsy ( *n* = 1; *m* = 1), hypersensitivity ( *n* = 1; *m* = 1), and muscle hemorrhage ( *n* = 1; *m* = 1).

b aPCC-related AEs for patients receiving aPCC in the regular volume at 2 U/kg/min were chills ( *n* = 1; *m* = 1), drug hypersensitivity ( *n* = 1; *m* = 1), headache ( *n* = 1; *m* = 2), hypersensitivity ( *n* = 1; *m* = 1), pruritus ( *n* = 1; *m* = 1), and urticaria ( *n* = 1; *m* = 1).

c aPCC-related AEs for patients receiving aPCC in the 50% reduced volume at 2 U/kg/min group were cough ( *n* = 1; *m* = 1), headache ( *n* = 2; *m* = 2), and increased fibrin D-dimer ( *n* = 1; *m* = 1).

d The aPCC-related AE for a patient receiving aPCC in the 50% reduced volume at 10 U/kg/min was increased systolic blood pressure ( *n* = 1; *m* = 1).

e AEs related to study procedures were chills ( *n* = 1; *m* = 1), drug hypersensitivity ( *n* = 1; *m* = 1), headache ( *n* = 3; *m* = 4), injection-site swelling ( *n* = 2; *m* = 2), and thrombophlebitis ( *n* = 1; *m* = 1).

#### AEs Related to Study Drug

In total, 12 AEs in nine patients (27.3%) were considered related to aPCC. In part 1 of the study, aPCC-related AEs were experienced by four patients (12.1%) receiving the regular volume at 2 U/kg/min and by four patients (13.3%) receiving the 50% reduced volume at 2 U/kg/min. In part 2 of the study, no AEs were considered related to aPCC with the 50% reduced volume at 4 U/kg/min. One patient (3.6%) receiving the 50% reduced volume at 10 U/kg/min had an aPCC-related AE (not related to administration).

#### AEs Related to Study Procedures

Nine AEs related to study procedures were reported in six patients (18.2%) during the study. In part 1 of the study, AEs related to study procedures were experienced by three patients (9.1%) receiving the regular volume at 2 U/kg/min and by four patients (13.3%) receiving the 50% reduced volume at 2 U/kg/min. No AEs related to study procedures were reported in part 2 of the study.

Local and systemic AEs and those that were temporally associated or potentially related are shown in Supplementary Table S2 .

#### Exploratory Outcome Measures

No clinically meaningful differences were observed for the TASI in FII activity levels up to 48 hours after infusion between the regular volume and the 50% reduced volume in part 1 ( *p* = 0.908; Supplementary Fig. S2A ) or between the increased rates of 4 U/kg/min and 10 U/kg/min in part 2 ( *p* = 0.411; Supplementary Fig. S2B ).

**Fig. 2:**
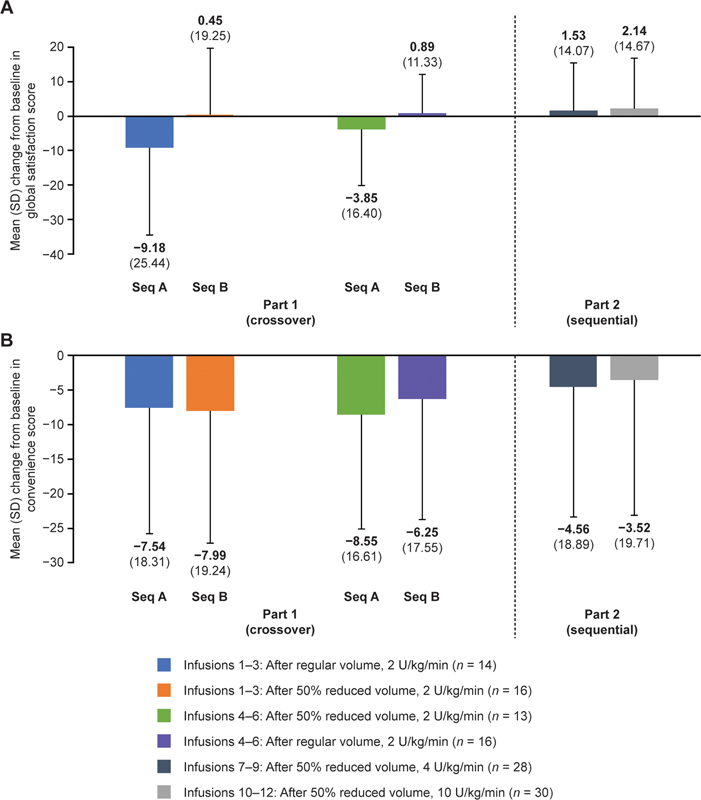
Summary of ( **A** ) global satisfaction and ( **B** ) convenience scores on the TSQM-9 (safety analysis set). Data are shown as the mean (SD) change from baseline in the score. Baseline is defined as the last nonmissing result before the first administration of aPCC. TSQM-9 scores were graded on a scale from 0 to 100, with higher scores indicating greater satisfaction. In part 1, patients in sequence A received aPCC in the regular volume first (infusions 1–3), then in the 50% reduced volume (infusions 4–6). Patients in sequence B received aPCC in the 50% reduced volume first (infusions 1–3), then in the regular volume (infusions 4–6). aPCC, activated prothrombin complex concentrate; *n* , number of patients; SD, standard deviation; TSQM-9, 9-item Treatment Satisfaction Questionnaire for Medication.

In part 1 of the study, there were more substantial decreases from baseline in TSQM-9 global satisfaction scores for sequence A than for sequence B after infusion 3 and before infusion 4 (mean [SD], A: −9.18 [25.44]; B: 0.45 [19.25]) and after infusion 6 and before infusion 7 (mean [SD], A: −3.85 [16.40]; B: 0.89 [11.33]; Fig. 2A ). In part 2 of the study, changes from baseline in mean TSQM-9 global satisfaction scores were similar when aPCC was administered in a 50% reduced volume at 4 U/kg/min and in a 50% reduced volume at 10 U/kg/min. In addition, decreases from baseline in mean TSQM-9 convenience scores were generally similar between sequence A and sequence B in part 1, as well as between the increased rates of 4 and 10 U/kg/min in part 2 ( Fig. 2B ).

At least 60% of patients reported in the patient preference questionnaire that they were “satisfied,” “very satisfied,” or “extremely satisfied” with the time taken to infuse aPCC throughout the study, with the highest satisfaction (82.1%) observed during part 2 after administration of aPCC in a 50% reduced volume at 4 U/kg/min ( Supplementary Fig. S3A ). Satisfaction levels for the ease of fitting the treatment into the patient's schedule were highest after administration of aPCC in a regular volume at 2 U/kg/min (78.6%) and after administration of aPCC in a 50% reduced volume at 4 U/kg/min (75.0%; Supplementary Fig. S3B ).

## Discussion

Management of bleeding in patients with hemophilia is significantly more challenging in those who develop inhibitors than in those who do not. 1 With the development of nonfactor therapies, the treatment landscape for patients with inhibitors is evolving; however, there is a continued need for BPAs (aPCC and rFVIIa) for the treatment of breakthrough bleeds and perioperative management. 1 In this study, we examined the safety and tolerability of administration of aPCC in a 50% reduced volume at the indicated infusion rate (2 U/kg/min at the time of the study) 12 and at faster infusion rates (4 and 10 U/kg/min) in patients with hemophilia A with inhibitors. Previous studies in patients with hemophilia with inhibitors have demonstrated that aPCC is well tolerated at the approved infusion volume and speed. 12 We found that aPCC was also well tolerated using the adapted dosing regimens, with safety profiles comparable to the approved infusion volume and speed.

Most AEs reported were mild or moderate in severity. Only two SAEs were considered either possibly or probably related to aPCC administration (drug hypersensitivity and hypersensitivity, respectively), both of which were reported in patients who received aPCC in a regular volume at 2 U/kg/min and led to study discontinuation. Both patients recovered from these SAEs. No thromboembolic events were reported during our study. This is consistent with previous studies showing that the incidence of thromboembolic events in patients receiving aPCC at the approved infusion volume and speed is very low (4–8 events per 100,000 infusions). 2 12 15 16 Moreover, global real-world evidence from 71 patients with congenital hemophilia with inhibitors demonstrated that a faster infusion rate of 3.8 U/kg/min, which is routinely used in clinical practice, did not increase the incidence of thromboembolic events. 13 The incidence of AHRs and ISRs observed in our study was also similar to real-world evidence of aPCC use. 2

Administration of aPCC using a reduced volume and faster infusion rate could help reduce the treatment burden for patients with hemophilia A with inhibitors, which is increased when compared with patients without inhibitors. 1 17 Treatment adherence has often represented a challenge in patients with hemophilia with inhibitors, due in part to the frequency of administration required for prophylaxis and the perceived burden of intravenous infusions. 1 Indeed, the transition from pediatric to adult care can exacerbate this perception, with increased infusion time arising from increased body weight and as patients develop the skills required for self-infusion. 1 A reduced infusion time also has clear potential benefits in acute clinical settings, such as on-demand treatment and surgical management. 13 In patients with inhibitors, even those who are receiving nonfactor therapy require BPAs for the treatment of breakthrough bleeds and the management of perioperative hemostasis. 1 During acute bleeding episodes in patients with hemophilia A with inhibitors, prompt treatment is critical. 1 Thus, a reduced time to administer aPCC through a reduced infusion volume and an increased infusion rate could result in an improved hemostatic effect, which for hemarthroses is associated with lower pressure in the bleeding joint, reduced iron deposition, and a decreased risk of cartilage destruction. 18

Hemophilia is associated with substantial psychological and economic burdens for patients and their caregivers. 19 In addition to reducing the treatment burden and improving adherence, optimizing the administration of aPCC could also benefit various other aspects of the lives of patients with hemophilia A with inhibitors, including the overall health-related quality of life of patients and caregivers. 1 20 21 During our study, more than 60% of patients reported that they were “satisfied,” “very satisfied,” or “extremely satisfied” with the time taken to infuse aPCC throughout the study. Patient satisfaction was relatively similar with different infusion volumes and rates, which may reflect the clinical trial setting.

aPCC promotes hemostasis through enhanced thrombin generation from FII (prothrombin). 22 23 Monitoring of pre- and postinfusion FII activity levels as a marker of coagulation was an exploratory objective of this study. No notable differences in the change in FII activity levels were observed between the different aPCC dosing regimens.

The limitations of this study include the short duration and lack of blinding to treatment. However, blinding was not practical owing to the inherent nature of the crossover study design and the need to infuse aPCC accurately at specific volumes and rates. It is also worth noting that patients who progressed to part 2 of the study were from a select group of patients who had received at least two of the three infusions in each sequence in part 1 and had reported no safety issues. In addition, this study considered only the global satisfaction and convenience domains of the TSQM-9 questionnaire. These domains were selected because they include questions that capture patients' unpleasant experiences with a medication and because the convenience domain has the strongest association with treatment adherence. 14 Although hematologic laboratory tests were performed to determine parameters such as fibrin D-dimer levels in our study, thrombin generation assays were not conducted.

The strengths of our study include the robust crossover design, which enabled four different combinations of infusion volumes and rates to be assessed; the prospective, multicenter nature of the study; use of the approved infusion volume and rate as a benchmark for safety profile data 12 ; and use of the TSQM-9 (an instrument that has previously been validated in patients with hemophilia A) to assess patient satisfaction. 24 Moreover, the time taken to infuse aPCC was shortened up to fivefold when administered at the infusion rate of 10 U/kg/min. Finally, our study addressed a key challenge in hemophilia A—the optimized treatment of patients with inhibitors—and has potential implications for improving treatment efficiency and satisfaction in clinical practice.

## Conclusions

In this study, aPCC was well tolerated when administered in a 50% reduced volume and at faster infusion rates (4 and 10 U/kg/min), with safety profiles similar to the approved infusion volume and rate. 12 Overall, these data provide support for the adaptation of aPCC administration, with the potential to reduce the treatment burden for patients with hemophilia A with inhibitors, as well as their caregivers and physicians.

## References

[1] WFH Guidelines for the Management of Hemophilia panelists and co-authors Srivastava A Santagostino E Dougall A WFH guidelines for the management of hemophilia, 3rd edition Haemophilia 2020 26 06 1 158 32744769 10.1111/hae.14046

[2] Perry D Berntorp E Tait C FEIBA prophylaxis in haemophilia patients: a clinical update and treatment recommendations Haemophilia 2010 16 01 80 89 10.1111/j.1365-2516.2009.02104.x 19780845

[3] Leissinger C A Prevention of bleeds in hemophilia patients with inhibitors: emerging data and clinical direction Am J Hematol 2004 77 02 187 193 15389908 10.1002/ajh.20162

[4] Kempton C L Meeks S L Toward optimal therapy for inhibitors in hemophilia Hematology Am Soc Hematol Educ Program 2014 2014 01 364 371 25696880 10.1182/asheducation-2014.1.364

[5] Ljung R Auerswald G Benson G Inhibitors in haemophilia A and B: management of bleeds, inhibitor eradication and strategies for difficult-to-treat patients Eur J Haematol 2019 102 02 111 122 30411401 10.1111/ejh.13193 PMC6936224

[6] DeKoven M Karkare S Lee W C Impact of haemophilia with inhibitors on caregiver burden in the United States Haemophilia 2014 20 06 822 830 25273645 10.1111/hae.12501

[7] Schultz N H Lundblad R Holme P A Activated prothrombin complex concentrate to reverse the factor Xa inhibitor (apixaban) effect before emergency surgery: a case series J Med Case Rep 2018 12 01 138 29764497 10.1186/s13256-018-1660-9 PMC5954448

[8] Novo Nordisk, Inc Summary of product characteristics, NovoSeven. European Medicines Agency 2006. Accessed October 24, 2023 at:https://www.ema.europa.eu/en/documents/product-information/novoseven-epar-product-information_en.pdf

[9] Novo Nordisk, Inc NOVOSEVEN ^®^ RT Coagulation Factor VIIa (Recombinant) . Prescribing information 2020. Accessed October 24, 2023 at:https://www.novo-pi.com/novosevenrt.pdf

[10] Laboratoire Français du Fractionnement et des Biotechnologies S.A. SEVENFACT ^®^ [coagulation factor VIIa (recombinant)-jncw] . Prescribing information 2022. Accessed October 24, 2023 at:https://www.fda.gov/media/136610/download

[11] Laboratoire Français du Fractionnement et des Biotechnologies S.A. Summary of product characteristics, CEVENFACTA. European Medicines Agency 2022. Accessed October 24, 2023 at:https://www.ema.europa.eu/en/documents/product-information/cevenfacta-epar-product-information_en.pdf

[12] Baxalta US, Inc., a Takeda company FEIBA ^®^ (anti-inhibitor coagulant complex) . Prescribing information 2020. Accessed August 04, 2023 at:https://www.shirecontent.com/PI/PDFs/FEIBA_USA_ENG.pdf

[13] FEIBA PASS Study group Negrier C Voisin S Baghaei F Global Post-Authorization Safety Surveillance Study: real-world data on prophylaxis and on-demand treatment using FEIBA (an activated prothrombin complex concentrate) Blood Coagul Fibrinolysis 2016 27 05 551 556 26829366 10.1097/MBC.0000000000000525 PMC4935538

[14] Bharmal M Payne K Atkinson M J Desrosiers M P Morisky D E Gemmen E Validation of an abbreviated Treatment Satisfaction Questionnaire for Medication (TSQM-9) among patients on antihypertensive medications Health Qual Life Outcomes 2009 7 36 19397800 10.1186/1477-7525-7-36 PMC2678998

[15] Ehrlich H J Henzl M J Gomperts E D Safety of factor VIII inhibitor bypass activity (FEIBA): 10-year compilation of thrombotic adverse events Haemophilia 2002 8 02 83 90 11952842 10.1046/j.1365-2516.2002.00532.x

[16] Antunes S V Tangada S Stasyshyn O Randomized comparison of prophylaxis and on-demand regimens with FEIBA NF in the treatment of haemophilia A and B with inhibitors Haemophilia 2014 20 01 65 72 10.1111/hae.12246 PMC4216433 23910578

[17] Oladapo A O Lu M Walsh S O'Hara J Kauf T L Inhibitor clinical burden of disease: a comparative analysis of the CHESS data Orphanet J Rare Dis 2018 13 01 198 30413215 10.1186/s13023-018-0929-9 PMC6230298

[18] FENOC Study Group Astermark J Donfield S M DiMichele D M A randomized comparison of bypassing agents in hemophilia complicated by an inhibitor: the FEIBA NovoSeven Comparative (FENOC) Study Blood 2007 109 02 546 551 16990605 10.1182/blood-2006-04-017988

[19] O'Hara J Hughes D Camp C Burke T Carroll L Diego D G The cost of severe haemophilia in Europe: the CHESS study Orphanet J Rare Dis 2017 12 01 106 28569181 10.1186/s13023-017-0660-y PMC5452407

[20] Thornburg C D Duncan N A Treatment adherence in hemophilia Patient Prefer Adherence 2017 11 1677 1686 29033555 10.2147/PPA.S139851 PMC5630068

[21] D'Angiolella L S Cortesi P A Rocino A The socioeconomic burden of patients affected by hemophilia with inhibitors Eur J Haematol 2018 101 04 435 456 29889317 10.1111/ejh.13108

[22] Turecek P L Varadi K Gritsch H Factor Xa and prothrombin: mechanism of action of FEIBA Vox Sang 1999 77 01 72 79 10529694 10.1159/000056722

[23] Turecek P L Váradi K Gritsch H Schwarz H P FEIBA: mode of action Haemophilia 2004 10 02 3 9 15385040 10.1111/j.1365-2516.2004.00934.x

[24] Sun H L Yang M Poon M C The impact of extended half-life factor concentrates on patient reported health outcome measures in persons with hemophilia A and hemophilia B Res Pract Thromb Haemost 2021 5 07 e12601 34667922 10.1002/rth2.12601 PMC8505988

