# Supplementary material for: Reduced Volume and Faster Infusion Rate of Activated Prothrombin Complex Concentrate: A Phase 3b/4 Trial in Adults with Hemophilia A with Inhibitors
Source: TH Open. 2024 Jul 8;8(3):e273–82. doi: 10.1055/s-0044-1787781 (PMC11230701; doi:10.1055/s-0044-1787781)
Supplement: Supplementary file 1 — Supplementary Material [file 10-1055-s-0044-1787781-s24020006.pdf]

Supplementary Material S1: Additional inclusion and exclusion criteria

- Each patient had to meet the following criteria to be eligible for the study.
  - Age ≥18 to ≤65 years at the time of screening.
  - Adequate venous access.
  - Willing and able to adhere to the requirements of the protocol.
  - In women of childbearing potential, a negative blood pregnancy test and agreed to employ adequate birth control measures for the duration of the study, such as the following.
    - Abstinence from sexual intercourse.
    - Using a reliable method of contraception (such as an intrauterine device, barrier method [e.g., diaphragm or sponge; female condom not permitted] with spermicide, oral contraceptive, injectable progesterone, subdermal implant) and having their male partner use a condom.
  - If a woman of nonchildbearing potential, status confirmed at screening by fulfilling one of the following criteria.
    - Postmenopausal, defined as amenorrhea for at least 12 months following cessation of all exogenous hormonal treatments and with follicle-stimulating hormone levels within the laboratory-defined postmenopausal range, or amenorrhea for at least 24 months and receiving hormonal replacement therapy.
    - Documentation of irreversible surgical sterilization by hysterectomy, bilateral oophorectomy, bilateral tubal ligation (with no subsequent pregnancy at least 1 year after bilateral tubal ligation), or bilateral salpingectomy.
- Patients who met any of the following criteria were excluded from the study.
  - Diagnosis of advanced atherosclerosis, malignancy, and/or other diseases that could increase the patient's risk of thromboembolic complications.
  - Taking any immunomodulating drug (e.g., corticosteroid agents at a dose equivalent to hydrocortisone >10 mg/day, or α-interferon) in the 30 days before enrollment, except antiretroviral chemotherapy.
  - Taking herbal supplements that contained antiplatelet activity.
  - Participation in another clinical study involving an investigational product or investigational device in the 30 days before enrollment or being scheduled to participate in another clinical study involving an investigational product or investigational device during the course of this study.
  - Being a family member or employee of the investigator.
  - Having a clinically significant medical, psychiatric, or cognitive illness, or recreational drug/alcohol use that, in the opinion of the investigator, would affect patient safety or compliance.

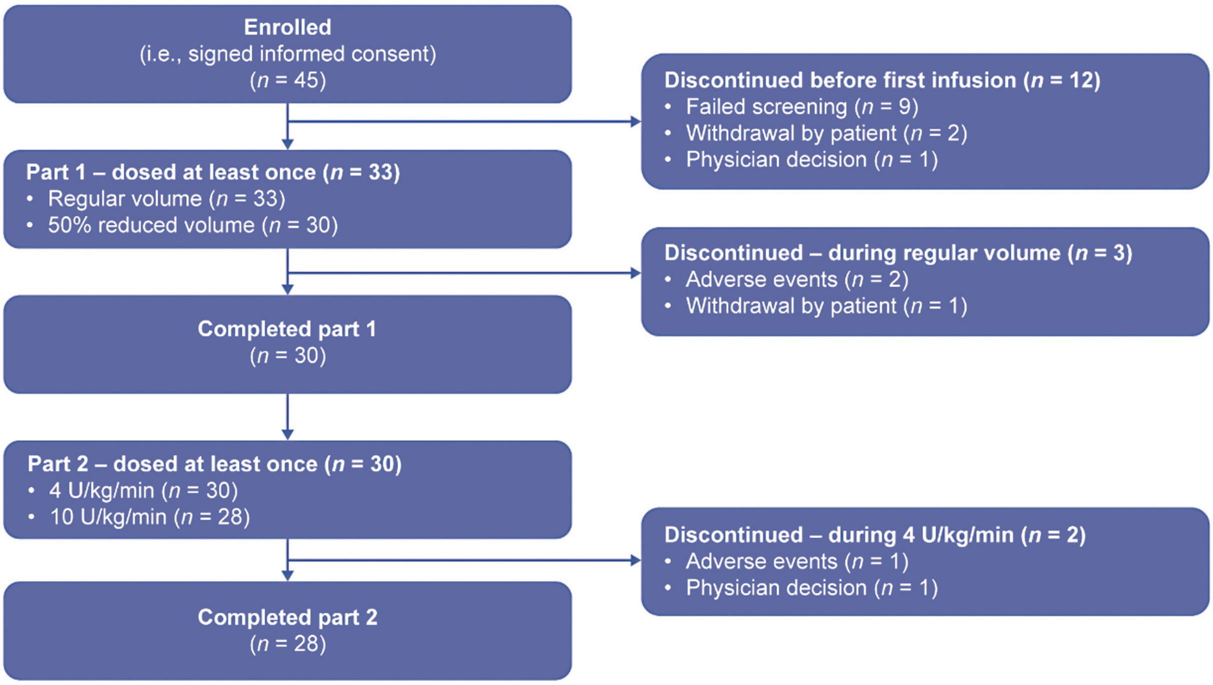

Supplementary Fig. S1 Patient disposition (CONSORT flow diagram). *n*, number of patients.

 © 2024. The Author(s).

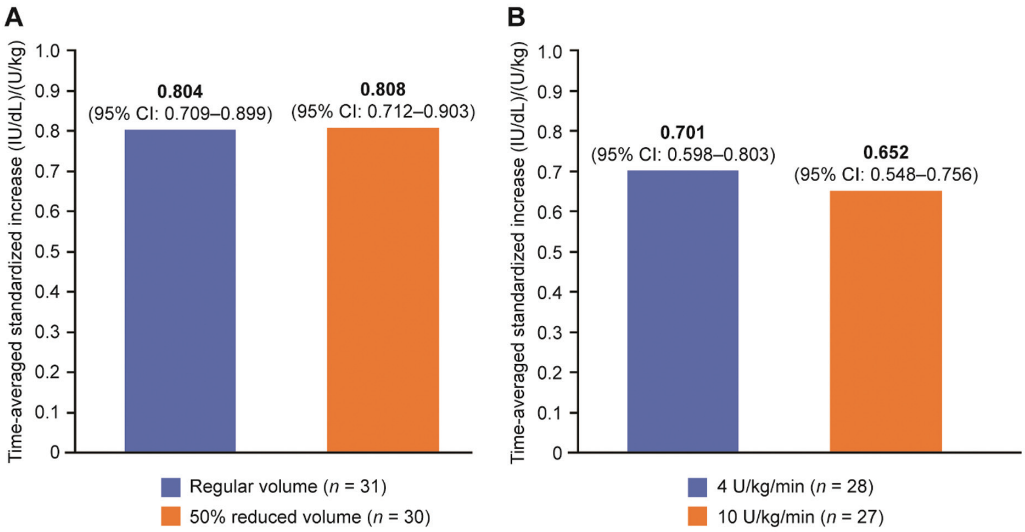

Supplementary Fig. S2 Time-averaged standardized increases (IU/dL)/(U/kg) in coagulation factor II activity levels. Comparisons of aPCC administered in **(A)** the regular versus 50% reduced volume at an infusion rate of 2 U/kg/min (part 1), and **(B)** the increased infusion rate of 4 U/kg/min versus 10 U/kg/min in a 50% reduced volume (part 2; full analysis set). Reported factor II values that were above the upper limit of quantification (i.e., >5 IU/mL) were treated as missing samples and were therefore not included in the analysis. aPCC, activated prothrombin complex concentrate; CI, confidence interval.

 © 2024. The Author(s).

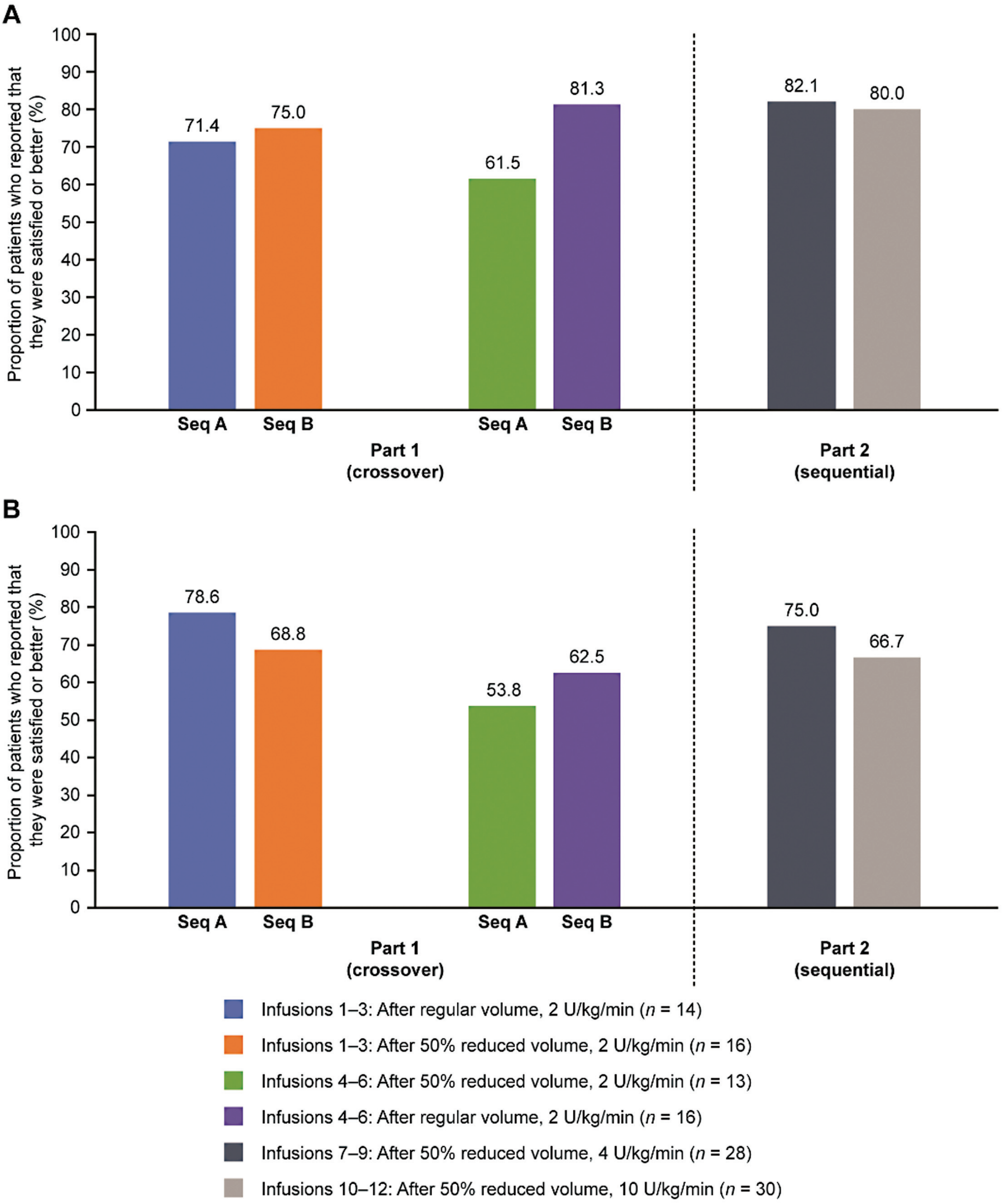

Supplementary Fig. S3 Summary of scores for **(A)** the time taken to infuse and **(B)** the ease of fitting treatment into patients' schedules from the patient preference questionnaire (safety analysis set). Proportions of patients for each category are expressed relative to the number of patients with a nonmissing result in the relevant analysis set. In part 1, patients in sequence A received aPCC in the regular volume first (infusions 1–3), then in the 50% reduced volume (infusions 4–6). Patients in sequence B received aPCC in the 50% reduced volume first (infusions 1–3), then in the regular volume (infusions 4–6). Responses were defined on a 7-point Likert satisfaction scale: extremely satisfied, very satisfied, satisfied, somewhat satisfied, dissatisfied, very dissatisfied, extremely dissatisfied. aPCC, activated prothrombin complex concentrate; *n*, number of patients.

 © 2024. The Author(s).

Supplementary Table S1 Summary of AEs by system organ class

AE, *n* (%) [*m*]	Regular volume 2U/kg/min (*n* = 33)	50% reduced volume 2U/kg/min (*n* = 30)	50% reduced volume 4U/kg/min (*n* = 30)	50% reduced volume 10U/kg/min (*n* = 28)	Overall 2U/kg/min (both volumes) (*n* = 33)	Overall 50% reduced volume (all infusion rates) (*n* = 30)	Overall (*n* = 33)
Musculoskeletal and connective tissue disorders	1 (3.0) [1]	3 (10.0) [5]	0 (0.0) [0]	2 (7.1) [2]	4 (12.1) [6]	4 (13.3) [7]	5 (15.2) [8]
Arthralgia	1 (3.0) [1]	3 (10.0) [4]	0 (0.0) [0]	2 (7.1) [2]	4 (12.1) [5]	4 (13.3) [6]	5 (15.2) [7]
Muscle hemorrhage	0 (0.0) [0]	1 (3.3) [1]	0 (0.0) [0]	0 (0.0) [0]	1 (3.0) [1]	1 (3.3) [1]	1 (3.0) [1]
General disorders and administration-site conditions	3 (9.1) [3]	1 (3.3) [2]	0 (0.0) [0]	0 (0.0) [0]	4 (12.1) [5]	1 (3.3) [2]	4 (12.1) [5]
Injection-site swelling	1 (3.0) [1]	1 (3.3) [1]	0 (0.0) [0]	0 (0.0) [0]	2 (6.1) [2]	1 (3.3) [1]	2 (6.1) [2]
Chills	1 (3.0) [1]	0 (0.0) [0]	0 (0.0) [0]	0 (0.0) [0]	1 (3.0) [1]	0 (0.0) [0]	1 (3.0) [1]
Feeling cold	0 (0.0) [0]	1 (3.3) [1]	0 (0.0) [0]	0 (0.0) [0]	1 (3.0) [1]	1 (3.3) [1]	1 (3.0) [1]
Pyrexia	1 (3.0) [1]	0 (0.0) [0]	0 (0.0) [0]	0 (0.0) [0]	1 (3.0) [1]	0 (0.0) [0]	1 (3.0) [1]
Nervous system disorders	2 (6.1) [3]	2 (6.7) [2]	0 (0.0) [0]	0 (0.0) [0]	4 (12.1) [5]	2 (6.7) [2]	4 (12.1) [5]
Headache	1 (3.0) [2]	2 (6.7) [2]	0 (0.0) [0]	0 (0.0) [0]	3 (9.1) [4]	2 (6.7) [2]	3 (9.1) [4]
Epilepsy	1 (3.0) [1]	0 (0.0) [0]	0 (0.0) [0]	0 (0.0) [0]	1 (3.0) [1]	0 (0.0) [0]	1 (3.0) [1]
Infections and infestations	1 (3.0) [1]	0 (0.0) [0]	1 (3.3) [1]	1 (3.6) [1]	1 (3.0) [1]	2 (6.7) [2]	3 (9.1) [3]
Bronchitis	0 (0.0) [0]	0 (0.0) [0]	0 (0.0) [0]	1 (3.6) [1]	0 (0.0) [0]	1 (3.3) [1]	1 (3.0) [1]
Nasopharyngitis	1 (3.0) [1]	0 (0.0) [0]	0 (0.0) [0]	0 (0.0) [0]	1 (3.0) [1]	0 (0.0) [0]	1 (3.0) [1]
Viral infection	0 (0.0) [0]	0 (0.0) [0]	1 (3.3) [1]	0 (0.0) [0]	0 (0.0) [0]	1 (3.3) [1]	1 (3.0) [1]
Immune system disorders	2 (6.1) [2]	0 (0.0) [0]	0 (0.0) [0]	0 (0.0) [0]	2 (6.1) [2]	0 (0.0) [0]	2 (6.1) [2]
Drug hypersensitivity	1 (3.0) [1]	0 (0.0) [0]	0 (0.0) [0]	0 (0.0) [0]	1 (3.0) [1]	0 (0.0) [0]	1 (3.0) [1]
Hypersensitivity	1 (3.0) [1]	0 (0.0) [0]	0 (0.0) [0]	0 (0.0) [0]	1 (3.0) [1]	0 (0.0) [0]	1 (3.0) [1]
Investigations	0 (0.0) [0]	1 (3.3) [2]	0 (0.0) [0]	1 (3.6) [1]	1 (3.0) [2]	2 (6.7) [3]	2 (6.1) [3]
Increased systolic blood pressure	0 (0.0) [0]	0 (0.0) [0]	0 (0.0) [0]	1 (3.6) [1]	0 (0.0) [0]	1 (3.3) [1]	1 (3.0) [1]
Increased fibrin D-dimer	0 (0.0) [0]	1 (3.3) [1]	0 (0.0) [0]	0 (0.0) [0]	1 (3.0) [1]	1 (3.3) [1]	1 (3.0) [1]
SARS-CoV-2 antibody test positive	0 (0.0) [0]	1 (3.3) [1]	0 (0.0) [0]	0 (0.0) [0]	1 (3.0) [1]	1 (3.3) [1]	1 (3.0) [1]
Respiratory, thoracic, and mediastinal disorders	1 (3.0) [2]	1 (3.3) [1]	0 (0.0) [0]	0 (0.0) [0]	2 (6.1) [3]	1 (3.3) [1]	2 (6.1) [3]
Cough	1 (3.0) [1]	1 (3.3) [1]	0 (0.0) [0]	0 (0.0) [0]	2 (6.1) [2]	1 (3.3) [1]	2 (6.1) [2]
Hemoptysis	1 (3.0) [1]	0 (0.0) [0]	0 (0.0) [0]	0 (0.0) [0]	1 (3.0) [1]	0 (0.0) [0]	1 (3.0) [1]
Injury, poisoning, and procedural complications	0 (0.0) [0]	1 (3.3) [1]	0 (0.0) [0]	0 (0.0) [0]	1 (3.0) [1]	1 (3.3) [1]	1 (3.0) [1]

 © 2024. The Author(s).

Supplementary Table S1 (*Continued*)

AE, *n* (%) [*m*]	Regular volume 2U/kg/min (*n* = 33)	50% reduced volume 2U/kg/min (*n* = 30)	50% reduced volume 4U/kg/min (*n* = 30)	50% reduced volume 10U/kg/min (*n* = 28)	Overall 2U/kg/min (both volumes) (*n* = 33)	Overall 50% reduced volume (all infusion rates) (*n* = 30)	Overall (*n* = 33)
Fall	0 (0.0) [0]	1 (3.3) [1]	0 (0.0) [0]	0 (0.0) [0]	1 (3.0) [1]	1 (3.3) [1]	1 (3.0) [1]
Skin and subcutaneous tissue disorders	1 (3.0) [2]	0 (0.0) [0]	0 (0.0) [0]	0 (0.0) [0]	1 (3.0) [2]	0 (0.0) [0]	1 (3.0) [2]
Pruritus	1 (3.0) [1]	0 (0.0) [0]	0 (0.0) [0]	0 (0.0) [0]	1 (3.0) [1]	0 (0.0) [0]	1 (3.0) [1]
Urticaria	1 (3.0) [1]	0 (0.0) [0]	0 (0.0) [0]	0 (0.0) [0]	1 (3.0) [1]	0 (0.0) [0]	1 (3.0) [1]
Vascular disorders	0 (0.0) [0]	1 (3.3) [1]	0 (0.0) [0]	0 (0.0) [0]	1 (3.0) [1]	1 (3.3) [1]	1 (3.0) [1]
Thrombophlebitis	0 (0.0) [0]	1 (3.3) [1]	0 (0.0) [0]	0 (0.0) [0]	1 (3.0) [1]	1 (3.3) [1]	1 (3.0) [1]

Abbreviations: AE, adverse event; *m*, number of events; *n*, number of patients; SARS-CoV-2, severe acute respiratory syndrome coronavirus 2.

Supplementary Table S2 Additional safety outcome measures (safety analysis set)

AE, *n* (%) [*m*]	Regular volume 2 U/kg/min (*n* = 33)	50% reduced volume 2 U/kg/min (*n* = 30)	50% reduced volume 4 U/kg/min (*n* = 30)	50% reduced volume 10 U/kg/min (*n* = 28)	Overall 2 U/kg/min (both volumes) (*n* = 33)	Overall 50% reduced volume (all infusion rates) (*n* = 30)	Overall (*n* = 33)
Local[a]	1 (3.0) [1]	1 (3.3) [2]	0 (0.0) [0]	0 (0.0) [0]	2 (6.1) [3]	1 (3.3) [2]	2 (6.1) [3]
Systemic[b]	7 (21.2) [13]	7 (23.3) [12]	1 (3.3) [1]	4 (14.3) [4]	13 (39.4) [25]	10 (33.3) [17]	15 (45.5) [30]
Temporally associated[c]	7 (21.2) [10]	6 (20.0) [7]	0 (0.0) [0]	0 (0.0) [0]	12 (36.4) [17]	6 (20.0) [7]	12 (36.4) [17]
Temporally associated or potentially related[d]	7 (21.2) [10]	6 (20.0) [7]	0 (0.0) [0]	1 (3.6) [1]	12 (36.4) [17]	7 (23.3) [8]	13 (39.4) [18]

Abbreviations: AE, adverse event; *m*, number of events; *n*, number of patients; SARS-CoV-2, severe acute respiratory syndrome coronavirus 2.

[a]Local AEs were fall ($n = 1$; $m = 1$) and injection-site swelling ($n = 2$; $m = 2$).

[b]Systemic AEs were arthralgia ($n = 5$; $m = 7$), bronchitis ($n = 1$; $m = 1$), chills ($n = 1$; $m = 1$), cough ($n = 2$; $m = 2$), drug hypersensitivity ($n = 1$; $m = 1$), epilepsy ($n = 1$; $m = 1$), feeling cold ($n = 1$; $m = 1$), headache ($n = 3$; $m = 4$), hemoptysis ($n = 1$; $m = 1$), hypersensitivity ($n = 1$; $m = 1$), increased fibrin D-dimer ($n = 1$; $m = 1$), increased systolic blood pressure ($n = 1$; $m = 1$), muscle hemorrhage ($n = 1$; $m = 1$), nasopharyngitis ($n = 1$; $m = 1$), pruritus ($n = 1$; $m = 1$), pyrexia ($n = 1$; $m = 1$), SARS-CoV-2 antibody test result positive ($n = 1$; $m = 1$), thrombophlebitis ($n = 1$; $m = 1$), urticaria ($n = 1$; $m = 1$), and viral infection ($n = 1$; $m = 1$).

[c]Temporally associated AEs were defined as AEs that began during infusion or in the 24 hours (or 1 day if the time of onset was not available) after completion of infusion, irrespective of being related or not related to treatment. These were arthralgia ($n = 1$; $m = 1$), chills ($n = 1$; $m = 1$), cough ($n = 1$; $m = 1$), drug hypersensitivity ($n = 1$; $m = 1$), epilepsy ($n = 1$; $m = 1$), headache ($n = 3$; $m = 4$), hypersensitivity ($n = 1$; $m = 1$), increased fibrin D-dimer ($n = 1$; $m = 1$), injection-site swelling ($n = 2$; $m = 2$), pruritus ($n = 1$; $m = 1$), pyrexia ($n = 1$; $m = 1$), thrombophlebitis ($n = 1$; $m = 1$), and urticaria ($n = 1$; $m = 1$).

[d]Temporally associated or potentially related AEs were defined as AEs that began during infusion or in the 24 hours (or 1 day if the time of onset was not available) after completion of infusion; AEs considered by the investigator and/or the sponsor to be possibly or probably related to the study drug; or AEs for which causality assessment was missing. These were arthralgia ($n = 1$; $m = 1$), chills ($n = 1$; $m = 1$), cough ($n = 1$; $m = 1$), drug hypersensitivity ($n = 1$; $m = 1$), epilepsy ($n = 1$; $m = 1$), headache ($n = 3$; $m = 4$), hypersensitivity ($n = 1$; $m = 1$), increased fibrin D-dimer ($n = 1$; $m = 1$), increased systolic blood pressure ($n = 1$; $m = 1$), injection-site swelling ($n = 2$; $m = 2$), pruritus ($n = 1$; $m = 1$), pyrexia ($n = 1$; $m = 1$), thrombophlebitis ($n = 1$; $m = 1$), and urticaria ($n = 1$; $m = 1$).

 © 2024. The Author(s).
